# Structure of a Chaperone-Usher Pilus Reveals the Molecular Basis of Rod Uncoiling

**DOI:** 10.1016/j.cell.2015.11.049

**Published:** 2016-01-14

**Authors:** Manuela K. Hospenthal, Adam Redzej, Karen Dodson, Marta Ukleja, Brandon Frenz, Catarina Rodrigues, Scott J. Hultgren, Frank DiMaio, Edward H. Egelman, Gabriel Waksman

**Affiliations:** 1 Institute of Structural and Molecular Biology, University College London and Birkbeck, Malet Street, London, WC1E 7HX, UK; 2 Department of Biochemistry, University of Washington, Seattle, WA 98105, USA; 3 Center for Women’s Infectious Disease Research and Department of Molecular Microbiology, Washington University School of Medicine, St. Louis, MO 63011, USA; 4 Department of Biochemistry and Molecular Genetics, University of Virginia, Charlottesville, VA 22901, USA

## Abstract

Types 1 and P pili are prototypical bacterial cell-surface appendages playing essential roles in mediating adhesion of bacteria to the urinary tract. These pili, assembled by the chaperone-usher pathway, are polymers of pilus subunits assembling into two parts: a thin, short tip fibrillum at the top, mounted on a long pilus rod. The rod adopts a helical quaternary structure and is thought to play essential roles: its formation may drive pilus extrusion by preventing backsliding of the nascent growing pilus within the secretion pore; the rod also has striking spring-like properties, being able to uncoil and recoil depending on the intensity of shear forces generated by urine flow. Here, we present an atomic model of the P pilus generated from a 3.8 Å resolution cryo-electron microscopy reconstruction. This structure provides the molecular basis for the rod’s remarkable mechanical properties and illuminates its role in pilus secretion.

## Highlights

• The atomic structure of a chaperone-usher pilus rod was solved by cryo-EM
• Each subunit makes contact with five preceding and five succeeding subunits
• Mutations at subunit-subunit interfaces affect rod formation, not polymerization
• The structure elucidates the molecular basis for rod uncoiling

## Graphical Abstract

**Figure:**
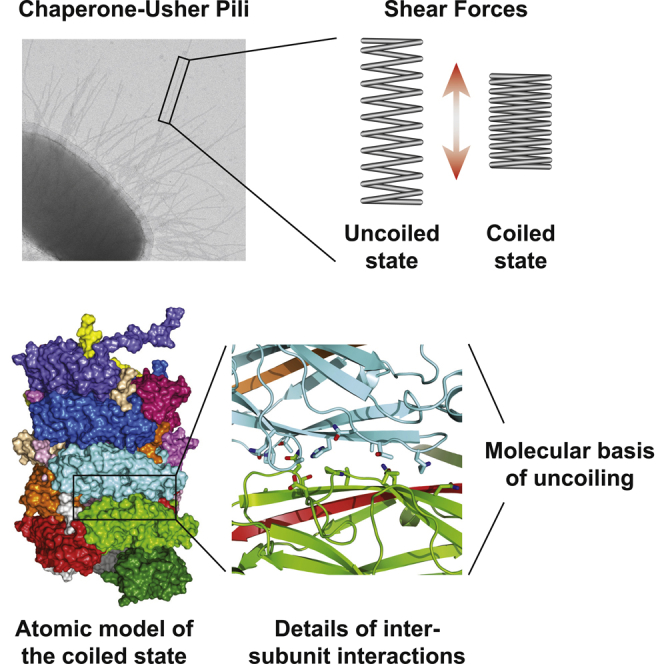

## Teaser

An atomic model of the P pilus rod generated from a 3.8 Å resolution cryo-EM reconstruction provides the molecular basis for its remarkable mechanical properties that allow bacteria to maintain adhesion to the urinary tract.

## Introduction

**Figure S1:**
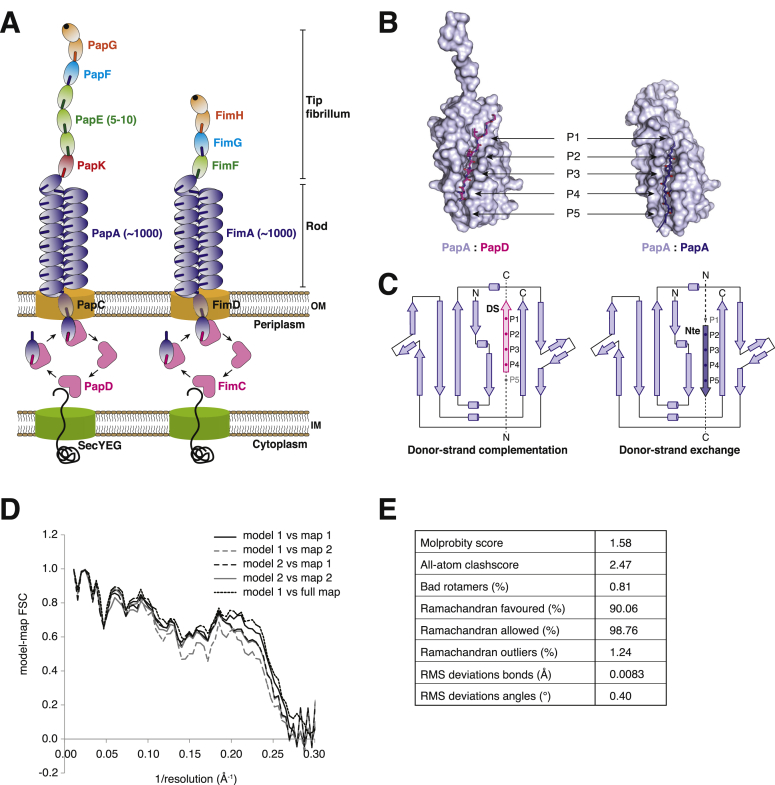
Architecture of Type 1 and P Pili, Their Assembly Mechanism via Donor-Strand Complementation and Donor-Strand Exchange, and Model Building and Refinement of the P Pilus Rod, Related to Figure 1 (A) Pilus subunits are transported to the periplasm through the SecYEG transporter in the inner membrane (IM), where a chaperone (FimC or PapD) assists in the folding and transport of subunits to the usher situated in the outer membrane (OM). Here, the subunits polymerize and are assembled into a pilus which can be divided into a thin ‘tip fibrillum’ and a ‘helically wound rod’. (B) Subunits are incorporated into the growing pilus through sequential steps of donor-strand exchange (DSE). The subunits are unstable on their own, as they consist of C-terminally truncated Ig-folds lacking strand G. As a result of the missing strand, a large hydrophobic groove is created where the strand G would have been if the fold had been complete. As they emerge from the SecYEG transporter, subunits are captured by the chaperone, which inserts its G1 β strand into the hydrophobic groove of the subunit thereby completing and stabilizing its fold. This is termed donor-strand complementation (DSC). The chaperone’s P1 to P4 residues are positioned in the subunits groove’s P1 to P4 pockets. The P5 pocket remains empty in DSC. In the pilus, the N-terminal extension (Nte; 10-20 residues) of each subunit provides the ‘complementing’ β strand and is thus inserted into the preceding subunit’s groove, thereby stabilizing it structurally. This is termed donor-strand exchange (DSE). The transition from DSC to DSE occurs via a zip-in-zip-out mechanism whereby the Nte of the incoming subunit occupies the previously empty P5 pocket, before inserting into the P4, P3, P2 and P1 pockets. (C) Topology diagrams of a pilus subunit during DSC and DSE. The key difference is the orientation of the inserted β strand, in DSC the chaperone’s β strand is inserted in a parallel fashion, whereas in DSE the subunit’s Nte is inserted in a more stable anti-parallel fashion. The P1-P5 pockets are indicated by filled circles, note that the P5 pocket is vacant during DSC. DS, donor strand. (D) Two independent reconstructions (*map1* and *map2*) were used to guide refinement: models were refined against each, and evaluated against both the reconstruction used for refinement, as well as the independent reconstruction. These results indicate that the models are not overfit to the data: the difference in the agreement of models fit to *map1* compared to *map2* is explained by a relatively lower quality of *map2*. Finally, the agreement of the model fit to *map1* and the full map confirms the claimed resolution of 3.8 Å. (E) Model validation statistics.

Chaperone-usher (CU) pili are ubiquitous appendages displayed on the surface of bacterial pathogens (Thanassi et al., 1998). They play crucial roles in infection, being responsible for recognition and adhesion to host tissues. Types 1 and P pili are archetypal CU pili produced by uropathogenic *Escherichia coli* (UPEC) that mediate host-pathogen interactions critical in disease and biofilm formation (Flores-Mireles et al., 2015). Types 1 and P pili are composed of a short tip fibrillum made of three to four different subunits (FimH, FimG, and FimF for type 1 pili and PapG, PapF, PapE, and PapK for P pili) mounted on a 1–2 μM long and helically wound rod, which is composed of ∼1,000 copies of the major pilus subunit FimA or PapA for type 1 or P pili, respectively (Figure S1A) (Allen et al., 2012, Waksman and Hultgren, 2009).

Assembly of CU pili requires the assistance of two proteins: an outer-membrane (OM)-embedded assembly nanomachine termed the “usher” (FimD and PapC for type 1 and P pili, respectively) and a dedicated periplasmic chaperone (FimC and PapD for type 1 and P pili, respectively). The chaperone captures pilus subunits at the exit of the SecYEG inner-membrane transporter and facilitates their folding. Subunits by themselves lack all of the necessary steric information for folding, as they form C-terminally truncated Ig folds lacking strand G (Choudhury et al., 1999, Sauer et al., 1999, Vetsch et al., 2004). As a result of the missing strand, a deep longitudinal groove is observed on the subunit’s surface (Figure S1B). The chaperone “donates” one of its own strands to transiently complete the Ig fold of the subunit in a process termed donor-strand complementation (DSC) (Barnhart et al., 2000, Vetsch et al., 2004). Chaperone:subunit complexes then dock to the OM usher where polymerization occurs, and the nascent pilus is secreted. Polymerization at the usher occurs via a mechanism termed “donor-strand exchange” (DSE) (Figures S1B and S1C) (Sauer et al., 2002, Zavialov et al., 2003). During DSE, the donor strand provided by the chaperone to complement the subunit fold is replaced by another subunit’s N-terminal extension (Nte), a 10–20 residue extension found at the N terminus of each subunit except the subunit located at the very tip.

The usher catalyzes DSE by positioning all components of the DSE reaction in close proximity, thereby increasing the rate of reaction by several orders of magnitude (Nishiyama et al., 2008). The usher contains five domains: an N-terminal domain (NTD) that forms the primary recruitment site for chaperone:subunit complexes, a translocation pore through which the nascent pilus passes, a plug domain, and two C-terminal domains (CTDs) that form a secondary chaperone:subunit binding site (Geibel et al., 2013, Phan et al., 2011). In the resting state of the usher, the plug domain is located inside the pore. Upon engagement of the first subunit in assembly, the adhesin, the plug domain transitions to the periplasm next to the NTD, while the subunit inserts its lectin domain within the usher pore. In this activated form, the chaperone:adhesin complex is bound to the CTDs. Pilus subunits are then added sequentially via the following subunit incorporation cycle: (1) the chaperone:subunit complex next in assembly is recruited to the NTD; (2) this positions the Nte of the incoming subunit next to the groove of the subunit located at the CTDs; (3) DSE occurs, leading to the nascent pilus length increasing by one subunit and also leading to the dissociation of the chaperone in the chaperone:subunit complex located at the CTDs; (4) the CTDs site is now free, and the nascent pilus can transfer from the NTD to the CTDs. The nascent pilus translocates within the usher pore, progressively emerging on the other side of the membrane.

The origin of the forces and energy driving the translocation step is still unknown. The bacterial periplasm is devoid of ATP, and there is no chemical gradient on either side of the OM (Jacob-Dubuisson et al., 1994). The pilus rod subunit is known to form a polymer that undergoes a quaternary structural change as it extrudes from the usher pore, resulting in the formation of a helical filament (Bullitt and Makowski, 1995). It is believed that the formation of this helical structure powers the translocation step; however, in the absence of a pilus rod structure, this hypothesis remains to be tested.

Finally, the rod confers remarkable spring-like properties to the pilus as a whole (Fällman et al., 2005, Forero et al., 2006, Le Trong et al., 2010). Indeed, atomic force microscopy (AFM) experiments have demonstrated that the helical pilus rod can be subjected to reversible uncoiling. This ability of the pilus rod to uncoil under forces is thought to confer resistance to the high rate of urine flow known to occur in the urinary tract. The bacterium can thus maintain a foothold on the host, even in the presence of flow-induced shear forces.

Although extensive electron microscopy work has been carried out on CU pili, all were at very low resolution and, therefore, did not provide residue-specific atomic resolution details (Hahn et al., 2002, Mu and Bullitt, 2006). In a crucial step toward being able to test the various putative roles that the pilus rod has in driving translocation and in mediating mechanical resistance to external forces, we solved the 3.8 Å resolution structure of the P pilus rod using cryoelectron microscopy (cryo-EM).

## Results and Discussion

### General Architecture of the P Pilus Rod

**Figure 1:**
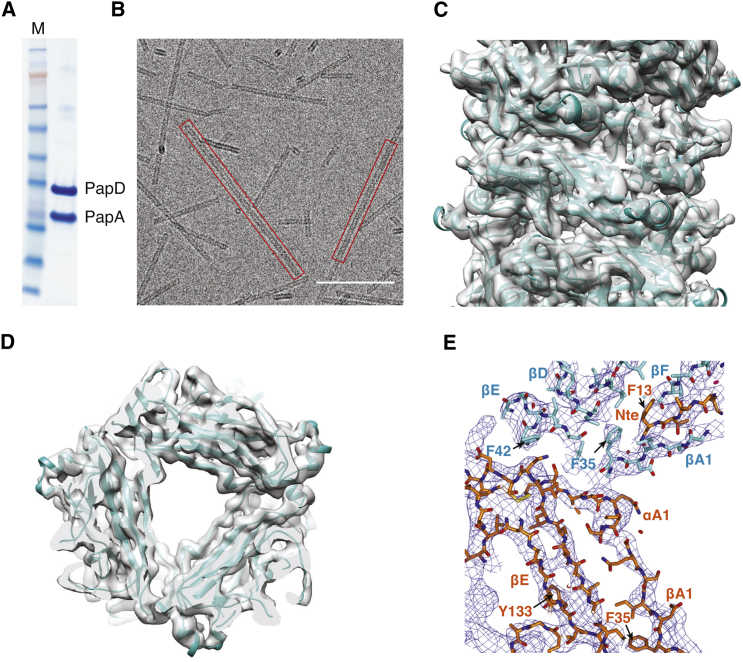
Purification of PapD:PapA and Electron Microscopy of the P Pilus Rod (A) SDS-PAGE of the purified PapD:PapA complex. M, molecular weight markers. (B) Electron micrograph of P pilus rods. Red rectangles indicate pilus rods. Scale bar, 100 nm. (C) Side-view of the experimentally derived electron density of the P pilus rod. The density was contoured at a 1.5 σ level and is shown as a semi-transparent surface colored in gray. A ribbon diagram of the refined atomic model is shown in cyan. (D) Top-view of the experimentally derived electron density. Density and model are as in (C). (E) Details of a representative region of the experimentally derived electron density. Electron density contoured at a 1.5 σ level is shown in chicken wire representation colored in blue. Only two PapA subunits of the final model are shown in stick representation with carbon atoms colored either in cyan or orange, while all oxygen and nitrogen atoms are colored in red and blue, respectively. Secondary structural elements are indicated as well as some side chains.

P pilus rods were produced in vitro from the purified PapD:PapA complex (Figure 1A). After their assembly, the rods were purified from unpolymerized PapD:PapA complexes by ultracentrifugation, applied to grids, and vitrified for cryo-EM analysis (Figure 1B). Data collection and structure determination proceeded as described in the Experimental Procedures. The resulting electron density displayed clear secondary structure elements and some side chains, in which a model of PapA with proper stereochemistry could be unambiguously built and refined (Figures 1C–1E, S1D, and S1E), providing the atomic structure of a CU pilus rod.

**Figure 2:**
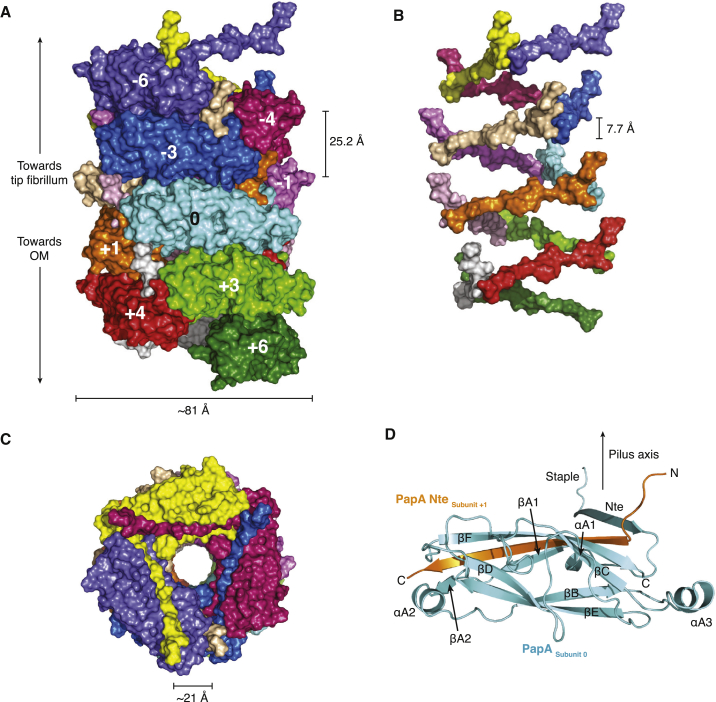
Structure of the P Pilus Rod (A) Surface diagram of the rod. Each subunit is shown in surface representation, color coded differently. The rod is oriented in such a way that the N termini of each subunit (the staples) are directed toward the top. In that orientation, the OM and tip fibrillum are toward the bottom and top, respectively. The subunit in cyan is the reference subunit and is numbered “0.” Subunits above or below this subunit are assembled before or after subunit 0, respectively, and are therefore numbered negatively (−1 to −6) or positively (+1 to +6), respectively. (B) Surface diagram focusing on the Ntes. The orientation of the pilus rod structure and the colorcoding of subunits are the same as in (A), but only the Ntes are shown, clearly illustrating the ascending path that the Ntes form within the structure. The rise from one subunit to another is indicated. (C) Top view of the pilus rod. The rod is represented as in (A). The Nte of the last subunit (−6) has been removed for clarity. (D) Ribbon diagram of the structure of PapA in the rod (subunit 0) in donor-strand exchange with the subunit next in assembly (subunit +1). The subunit is shown in cyan (labeled “PapA _subunit 0_”) with the Nte of subunit +1 colored in orange (labeled “PapA Nte _subunit +1_”). Secondary structure elements are labeled. The orientation of the subunit is the same as in (A). In that orientation, the staple extends approximately parallel to the pilus axis (indicated by an arrow).

The P pilus rod forms a helical filament of 3.28 subunits per turn, a pitch of 25.2 Å, and diameter of ∼81 Å. It contains a continuous central hollow lumen of ∼21 Å in diameter (Figures 2A–2C). In the orientation of the pilus shown in Figure 2A, the distal end of the pilus (the tip fibrillum) is located at the top, while the membrane-proximal end is at the bottom. The subunit colored in cyan serves as the reference subunit, termed “subunit 0.” Subunits above are labeled −1 to −6, since these subunits would have been assembled before subunit 0 during pilus biogenesis. Subunits below are labeled +1 to +6, because they would have been assembled subsequently. Strikingly, each subunit makes protein-protein interactions with ten other subunits, five preceding (−5 to −1) and five succeeding (+1 to +5).

### Structure of PapA Pre- and Post-insertion within the Rod

**Figure S2:**
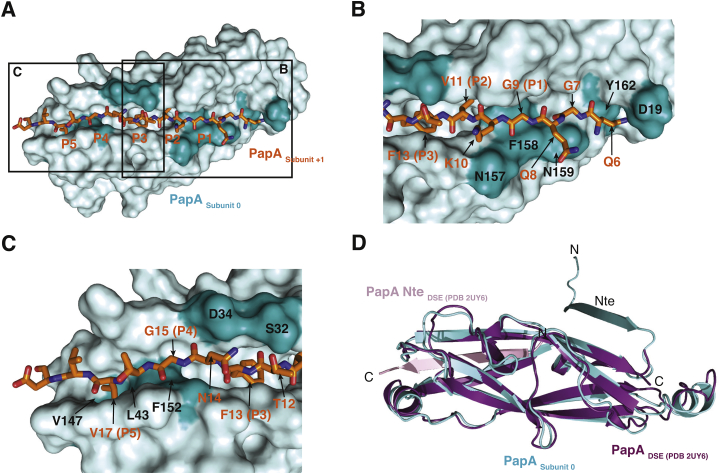
Interactions between PapA and Its Nte after Rod Formation and Superposition of PapA before and after Rod Insertion, Related to Figure 2 (A) Overview of the subunit-Nte interaction. The subunit and Nte are in cyan and orange respectively. Boxed areas labeled B and C locate the areas shown in panels B and C. Surfaces in dark teal locate residues mentioned in the main text. In panels B and C, residues in the subunit and in the Nte are labeled black and orange, respectively. The P1-P5 residues of the Nte are labeled both P1 to P5 and also by their residue numbering in the PapA sequence. The Nte of the subunit in cyan has been removed for clarity. (B) Area B of panel A. (C) Area C of panel A. (D) Superposition of the structure of PapA in the rod and that of PapA before rod formation. Both structures are shown in ribbon representation. PapA in the rod is labeled “PapA _subunit 0_” and is shown in cyan. PapA before rod formation is labeled “PapA _DSE (PDB__2UY6__)_” and is in magenta. The Nte depicted here is that inserted into PapA _DSE (PDB__2UY6__)_ as determined in the structure by Verger et al. (2007).

The structure of the PapA monomer could be built in its entirety (Figure 2D), as opposed to previous structures of PapD:PapA or PapA:PapA dimers that were incomplete (Verger et al., 2007). From residues 1–5, the very N-terminal end of PapA makes extensive stabilizing subunit-subunit contacts. This portion of PapA extends parallel to the rod axis and is termed the “staple” (Figure 2D) because of the multiple interfaces it makes with other subunits (see below). The complementing Nte strand starts from residue 6 and ends at residue 19 (Nte in Figure 2D) and inserts into the groove of the adjacent subunit. The Nte strand makes a sharp 90° angle with the staple region. This angle is imposed by Tyr162 that blocks the subunit groove and, thus, redirects the Nte strand away from the groove (Figures S2A and S2B). In addition, the first residue in the Nte, Gln6, interacts with the complemented subunit’s Nte, notably residue Asp19 (Figures S2A and S2B). This implies that Ntes of consecutive subunits form a continuous ascending path from the membrane to the tip fibrillum, each with a rise of ∼7.7 Å (Figure 2B).

In the first structures of CU pilus subunits bound to Nte strands (Remaut et al., 2006, Sauer et al., 2002), it was observed that the complementing Nte strand contained five alternating residues termed “P1–P5 residues” that insert into the subunit groove at regions or pockets, which were termed “P1–P5 pockets” (Figures S1 and S2A) (Remaut et al., 2006, Rose et al., 2008). In the PapA structure within the rod, these residues are observed making extensive interactions. Strikingly, two Gly residues in the Nte (Gly9 and Gly15, P1 and P4 residues, respectively) lie on top of two Phe residues (Phe158 and Phe152, respectively; Figures S2B and S2C). The Gly15-Phe152 interaction was thought to assist in registering the Nte within the receiving subunit’s groove, since no other residue’s side chain could be accommodated in this protruding region of the groove (Verger et al., 2007); it is now clear that two such constraints are imposed on the slotting of Nte strands within subunits’ grooves (no such constraints are imposed on the structure of the Nte in chaperone:subunit complexes). Other interactions include polar interactions with residues of the Nte facing out, between Gln8 or Lys10 of the Nte and Asn159 or Asn157, respectively (Figure S2B) or between Thr12 or Asn14 in the Nte strand and Ser32 and Asp34, respectively (Figure S2C).

The sequence after the Nte (from residue 20) forms the pilin fold, a fold previously characterized as a C-terminally truncated Ig-fold-lacking strand G. Compared to the structure of PapA in dimers (representing a state of PapA prior to incorporation in the rod), the structure of PapA in the rod has undergone a substantial conformational change in the region between strands βB and βC, where contacts with an adjacent subunit stabilize an extended region into a three-turn α-helix, αA3 (Figure S2D).

### P Pilus Rod Subunit-Subunit Interaction Network

The subunit-subunit interactions network within the pilus rod is strikingly large, with each subunit burying 45.5% of its total surface area. Such a large interaction network is at least in part due to the Nte, which not only extends far into the pilus structure, but also projects the staple region away from the subunit that it originates from and toward a region of the pilus where several subunits converge to make contact.

**Figure 3:**
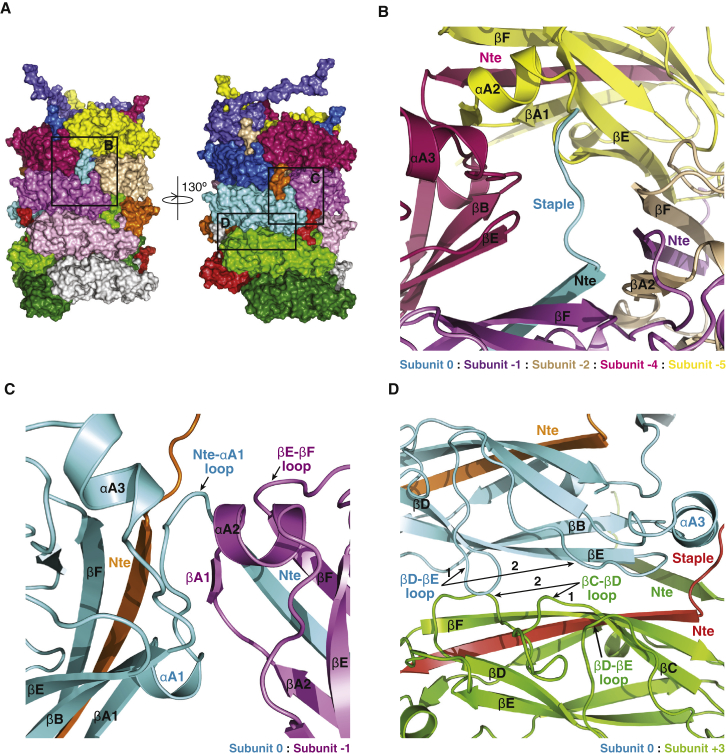
Details of Subunit-Subunit Interaction Interfaces (A) Surface diagram of the pilus rod and localization of the regions depicted in (B– D). Color coding and representation of subunits are as in Figure 2A. Black boxes labeled B, C, and D locate the region depicted in detail in panels (B–D). (B) Details of secondary structures involved in interactions between the staple of subunit 0 and subunits −1, −2, −4, and −5. Details of residues involved in these interactions are reported in Figures S3A and S3D. Subunits are in ribbon representation color coded as in (A). (C) Details of the secondary structures involved in interactions between subunits 0 and −1 in the region around the C-terminal part of the Nte and the Nte-αA1 loop of subunit 0. Representation and labeling are as in (B). Details of interacting residues are shown in corresponding Figures S3B and S3D. (D) Details of the secondary structures involved in interactions between subunits 0 and +3. Representation and labeling are as in (B). Details of interacting residues are shown in corresponding Figures S3C and S3D.

**Figure S3:**
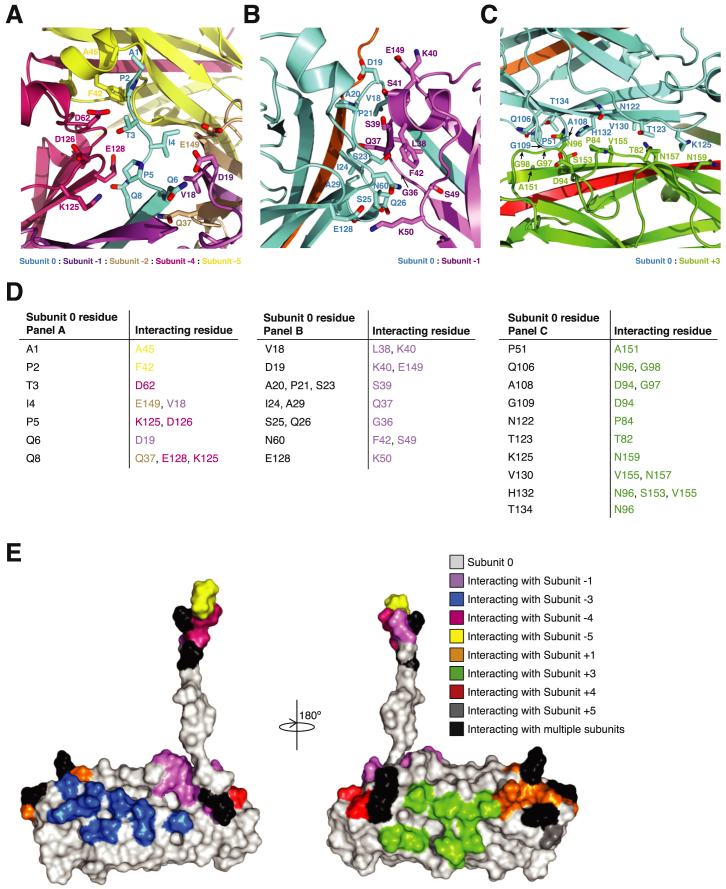
Details of the Interactions between Subunits within the Pilus Rod, Related to Figure 3 (A) Side chains involved in the interaction of adjacent subunits with the staple of subunit 0. Orientation and secondary structures are as in Figure 3B. At the very N terminus of the staple region, residues Ala1 and Pro2 (subunit 0) make interactions with Ala45 and Phe42 (subunit −5), respectively. Next, residue Thr3 (subunit 0) interacts with residue Asp62 (subunit −4), while Ile4 (subunit 0) interacts with Glu149 (subunit −2) and Val18 (subunit −1). Residue Pro5 (subunit 0) is in close proximity to Lys125 and Asp126 (subunit −4). Gln6 (subunit 0) is positioned at the interface between the donor-strand and the staple region and interacts with Asp19, which is positioned at the end of the complemented subunit’s Nte (subunit −1). Lastly Gln8 (subunit 0) interacts with Gln37 (subunit −2), as well as Glu128 and Lys125 (subunit −4). (B) Side chains involved in interactions between the C-terminal region of subunit 0’s Nte with subunit −1. Orientation and secondary structures are as in Figure 3C. Starting at the top of the panel, residue Asp19 (subunit 0) interacts with Glu149 and is also in close proximity to Lys40 (subunit −1). Next, Val18 (subunit 0) interacts with Leu38 and Lys40 (subunit −1). Residues Ala20, Pro21 and Ser23 (subunit 0) are in close proximity to Ser39 (subunit −1), while Ile24 and Ala29 (subunit 0) interact with Gln37 (subunit −1). In addition, both Ser25 and Gln26 (subunit 0) are positioned close to Gly36 (subunit −1), Residue Asn60 (subunit 0) makes contacts with both Phe42 and Ser49 (subunit −1). Lastly, Glu128 (subunit 0) makes a salt bridge interaction with Lys50 (subunit −1). (C) Side chains involved in interactions between subunit 0 and +3. Orientation and secondary structures are as in Figure 3D. Going from left to right, Gln106 (subunit 0) is positioned at the edge of the interface and is in close proximity to Asn96 and Gly98 (subunit +3). Next, Pro51 (subunit 0) interacts with Ala151 (subunit +3), while Thr134 (subunit 0) makes extensive contacts with Asn96 (subunit +3). Gly109 (subunit 0) interacts with Asp94 (subunit +3), while His132 (subunit 0) is crucial and makes contacts with Asn96, Ser153 and Val155 (subunit +3). Meanwhile, Ala108 (subunit 0) contacts Asp94 and Gly97 (subunit +3). Asn122 (subunit 0) interacts with Pro84 (subunit +3), while Val130 (subunit 0) interacts with Asn157 and is also in close proximity to Val155 (both of subunit +3). Lastly, Thr123 and Lys125 (subunit 0) interact with Thr82 and Asn159 (subunit +3), respectively. The main chains in panels A, B, and C are in ribbon representation while side chains are in stick representation with oxygen and nitrogen atoms color-coded in red and blue, respectively. Color-coding of ribbons and carbon atoms follows the color code established in Figure 2A for each subunit. (D) Table listing interactions shown in panels A, B, and C. (E) Surface mapping of subunit 0’s residues involved in interaction with other subunits. Color-coding indicates which subunits each residue is interacting with. The color code established in Figure 2A is used here. Residues colored in black represent regions which are interacting with multiple subunits.

Figure 3 provides an overview of the interactions that subunit 0 makes with other subunits. A detailed residue-specific description of these interactions is provided in Figure S3. Starting from the very N terminus, the staple region interacts with four subunits, subunits −1, −2, −4, and −5 (Figures 3B, S3A, S3D, and S3E). Overall, this region provides 347 Å^2^ of surface area to interactions between subunits. In contrast to the staple region, the Nte interacts mostly with the subunit that it complements (surface area buried: 1,138 Å^2^), and these interactions have been described above. The exception is Gln8, which interacts with residues in βE of subunit −4 (Figures 3B and S3A). Thus, while the very N-terminal end of the Nte is buried, the C-terminal end is exposed to the lumen (Figure 2C).

Figures 3C, S3B, and S3D describe the interactions in the region that follows the Nte. The loop region between Nte and αA1 of subunit 0 makes extensive interactions with βA1, αA2, and the βA1-αA2 loop of subunit −1. Of particular relevance are the main-chain-main-chain interactions between βA1 of subunit −1 and the loop between Nte and αA1 (main-chain atoms of Ser23 and Ile24) in subunit 0. Other relevant interactions include those between subunit 0’s βB-αA3 loop, αA3 residues, and subunit −1’s αA2 residues (for instance, Asn60 interacts with Phe42). Overall, this interface buries 408 Å^2^ of surface area.

The interfaces between subunit 0 and the subunits above and below (subunits −3 and +3, respectively) provide the bulk of the surface area involved in interactions outside of the Nte (buried surface area: 1,118 Å^2^). They are identical, and thus, only interactions between subunits 0 and +3 will be described here. The interactions are between residues in the βD-βE loop and in βE of subunit 0 and residues in the βC-βD loop, βD-βE loop, and in βF of subunit +3 (Figures 3D and S3C–S3E). Interactions involving the loops extend over large numbers of residues, but only two regions in each contribute to interactions (indicated as 1 and 2 in Figure 3D). Residues in region 1 of the βD-βE loop of subunit 0 interact with residues in region 2 of the βC-βD loop of subunit +3, while residues in region 2 of the βD-βE loop of subunit 0 interact both with residues in region 1 of the βC-βD loop and with residues in the βD-βE loop of subunit +3. Finally, subunit 0 also makes contact with the staple of subunit +4 and +5, with these interactions being identical to the interactions described in Figure 3B between subunit −4 or −5 and subunit 0.

Overall, the subunit-subunit interaction network that holds the rod’s quaternary structure together (i.e., all interactions except those involving the Nte) is polar. Most of the hydrophobic interactions are between complementing Nte and complemented groove residues. Thus, while the Nte-mediated interaction is strong (Puorger et al., 2008), the other interfaces between subunits are weaker, explaining why the rod can uncoil under shear forces without breaking apart.

### Structure Validation and Effect of Mutations at the Subunit-Subunit Interface

**Figure 4:**
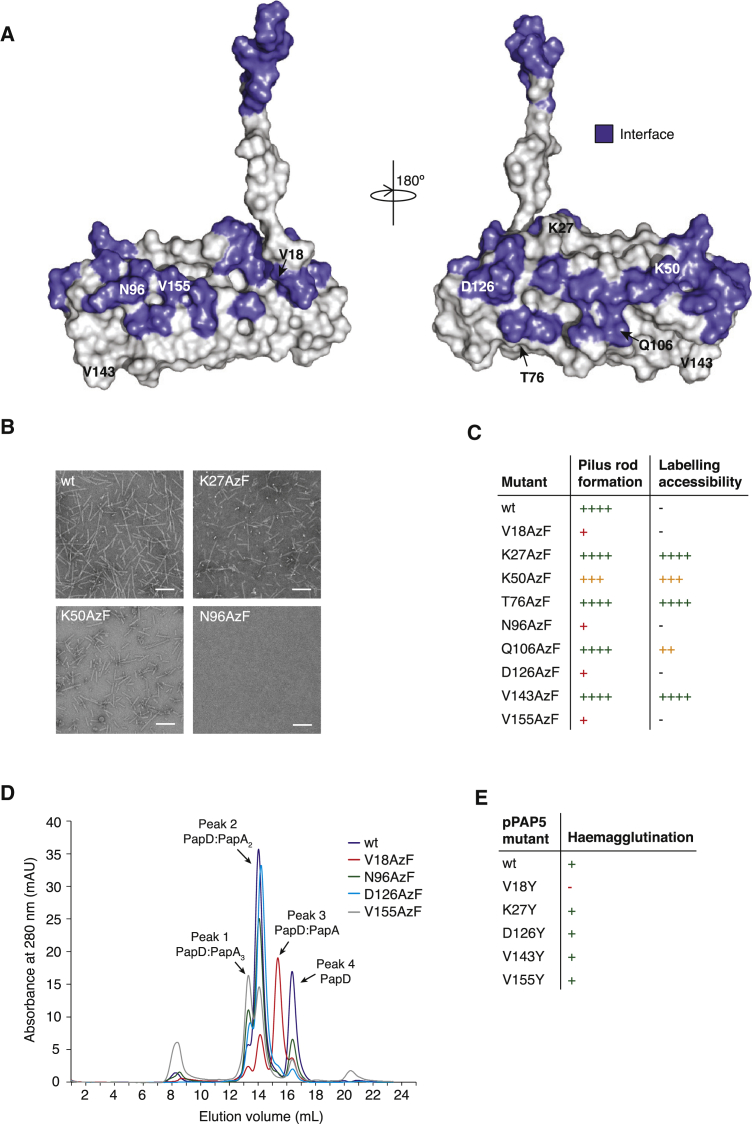
Probing the Structure by Site-Directed Mutagenesis and Site-Specific Labeling (A) Location of the residues targeted for site-directed incorporation of AzF. Surfaces in blue locate residues involved in subunit-subunit interactions as defined in Figures 3 and S3. (B) NS-EM of wild-type and mutant PapA rods. The full set of NS-EM micrographs is reported in Figure S4A. Here, only representative micrographs of three mutants are shown: one for a mutant not affected in pilus rod formation (Lys27), one for a mutant only partially affected in rod formation (Lys50), and one severely affected in rod formation (Asn96). Scale, 100 nm. (C) Summary of pilus rod formation and solvent accessibility of various residues within the rod structure. Each PapA variant is categorized and color coded according to its pilus rod formation and labeling efficiency. The quantification of these parameters is described in Experimental Procedures and the data are represented in graphical form in Figure S4B. Dash (-), no data available. (D) Size exclusion chromatography of mutants unable to form rods. The identity of each peak was evaluated by SEC-MALS (Figure S4C) and is indicated above the peak. (E) Summary of haemagglutination results (full results in Figure S4D). pPAP5 wild-type, untransformed HB101 cells (HB101 alone), and PBS served as controls for this experiment. All PapA mutants tested, with the exception of Val18Tyr, show a positive haemagglutination reaction with rabbit red blood cells.

Next, residues within, near, or outside of subunit-subunit interfaces were mutated. All three were required to provide validation of the rod structure. We mutated residues Val18, Lys27, Lys50, Thr76, Asn96, Gln106, Asp126, Val143, and Val155. The location of these residues is shown in Figure 4A. In the rod structure, Lys27, Thr76, and Val143 do not belong to any interface. Lys50 and Gln106 are on the edge of interaction surfaces (Figures S3B and S3C). Val18, Asn96, Asp126, and Val155 are within buried areas (Figures S3A–S3C).

These residues in PapA were chosen as sites for *p*-azido-L-phenylalanine (AzF) incorporation, using amber-suppression technology whereby this unnatural amino acid can be substituted at any place in the structure (see Experimental Procedures) (Chin et al., 2002). AzF substitutions present considerable advantages over other mutations: (1) AzF is similar to tyrosine and, thus, is just as effective in locally disrupting interfaces; yet (2) solvent accessibility of the mutated residue can be directly assessed by reacting an alkyne-derivatized fluorescent probe such as Alexa Fluor 647. Therefore, AzF site-directed mutagenesis is the ideal method to probe the validity of protein-protein complex structures and interfaces since it can probe both solvent-exposed surfaces and areas engaged in protein-protein interactions.

**Figure S4:**
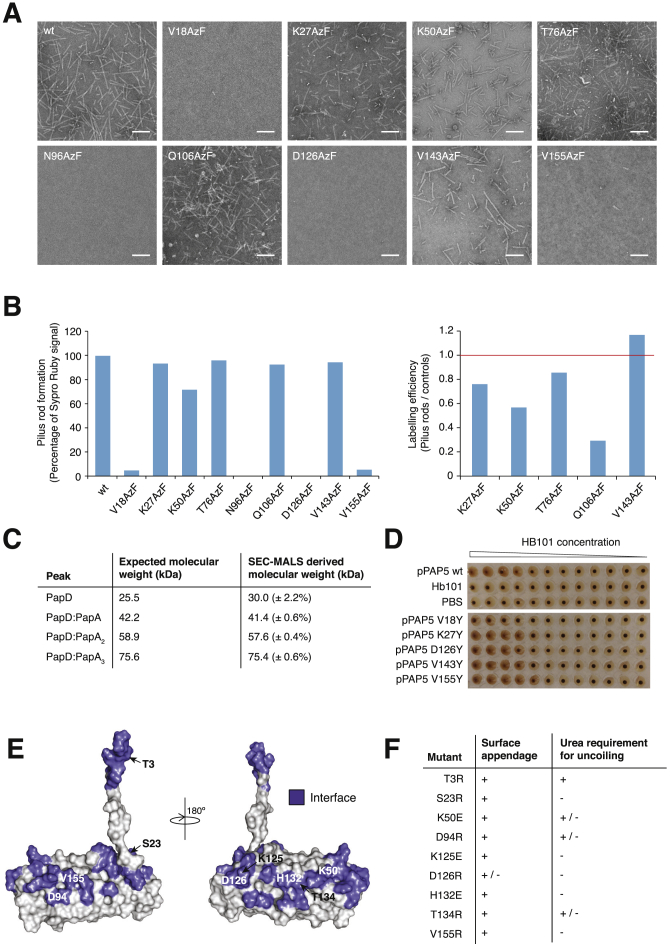
Biochemical Characterization of P Pilus Rod Mutants and In Vivo Assessment of Mutations of Interface Residues, Related to Figure 4 (A) The full set of negative stain images of all rod mutants after assembly into pilus rods and purification by ultracentrifugation. The scale bar represents 100 nm. (B) Quantification of pilus rod formation (left) and labeling efficiency (right). For pilus rod formation (left), the Sypro Ruby signal was converted into a percentage and normalized to the wild-type (100%). For labeling efficiency (right), if PapA in pilus rod samples labeled with Alexa 647 to the same extent as in the corresponding non-piliated control samples, the final ratio would be 1.0 (indicated by a red line). These data were used to categorise and color-code each PapA variant according to its pilus rod formation and labeling efficiency as shown in Figure 4C. For pilus rod formation, category 1 (+), 0%–29%; category 2 (++), 30%–59%; category 3 (+++), 60%–89%; category 4 (++++), > 90%. Dash (-), no data available. For labeling efficiency, category 1 (+), 0-0.25; category 2 (++), 0.26-0.5; category 3 (+++), 0.51-0.75; category 4 (++++), > 0.76. A detailed description of the quantitation can be found in the Experimental Procedures. The results presented in Figure 4C are from two independent experiments. (C) SEC-MALS results for each of the peaks reported for the PapD:PapA Val18AzF in Figure 4D and comparison to expected molecular weights. (D) Haemagglutination result of HB101 cells expressing the entire Pap operon (pPAP5 plasmid) with wild-type PapA or mutant versions of PapA. All pPAP5 PapA mutants showed a positive haemagglutination result, with the exception of Val18Tyr which haemagglutinated to a much lesser extent. Untransformed HB101 cells (HB101 alone) and PBS serve as negative controls. (E) Location of residues mutated in panel F. Representation is as in Figure 4A. (F) Summary of analysis of pilus translocation to the bacterial surface and assessment of the strength of helical interaction in the pilus. The residues mutated in this experiment are shown in panel E. The first column (surface appendage) indicates whether the PapA mutant has incorporated into pili displayed on the bacterial surface and the second column (urea requirement for uncoiling) is an assessment of the helical strength of these pili produced in vivo. (+) indicates results comparable to wild-type; (+/−) indicates either lesser pilus production and/or rod formation; (-) indicates either no pilus production or no rod formation. A description of the how these experiments were performed can be found in the Experimental Procedures section.

Two techniques were used to measure the impact of site-directed insertion of AzF on rod formation: (1) negative stain electron microscopy (NS-EM) to assess rod formation and (2) AzF labeling with Alexa Fluor 647 to assess solvent accessibility. The Lys27AzF, Thr76AzF, and Val143AzF mutants produce pili (Figures 4C, S4A, and S4B), and these positions are clearly solvent accessible (Figures 4C and S4B), consistent with their position in the rod structure. Labeling of the Lys50AzF and Gln106AzF mutants is decreased, confirming their position at the edge of the subunit-subunit interface, but only the Lys50AzF mutant is affected in rod formation (Figures 4B, 4C, S4A, and S4B).

The Val18AzF, Asn96AzF, Asp126AzF, and Val155AzF mutants could not be labeled, as they are greatly impaired in rod formation, as shown by NS-EM (Figures 4B and S4A), and thus, they could not be pelleted by ultracentrifugation. It could be that rod formation is abrogated, because these mutants are impaired in DSE in the first place. Thus, we tested whether the Val18AzF, Asn96AzF, Asp126AzF, and Val155AzF mutants could successfully undergo DSE. The PapA wild-type and mutants were left to polymerize as described in Experimental Procedures and were loaded on a size-exclusion chromatography column. For all samples (wild-type and mutants), three peaks were observed corresponding to PapD alone, PapD:PapA_2_, and PapD:PapA_3_, as assessed by size-exclusion chromatography-multi-angle light scattering (SEC-MALS) (Figures 4D and S4C). The elution profile of the mutants was compared to that of the wild-type and shown to be qualitatively identical except for the Val18 mutant, in which a substantial peak of unpolymerized PapD:PapA was observed (Figure 4D). Thus, the Asn96AzF, Asp126AzF, and Val155AzF mutants are able to undergo DSE but are impaired in rod quaternary structure formation, a predicted behavior given their central positions within the interfaces in which they participate. For the Val18AzF mutant, rod production is abrogated, because it is affected in its ability to undergo DSE. This is not surprising, as Val18 is part of the Nte, and Nte mutations have been shown to greatly disrupt this process (Remaut et al., 2006).

### Biological Impact of Mutations Affecting the Quaternary Structure of the Rod

Next, to ascertain whether mutants affected in rod formation are able to elaborate pili on the bacterial cell surface, haemagglutination assays were performed (details in Experimental Procedures) (Leffler and Svanborg-Edén, 1981). From the mutants described above, two affected in rod formation but still able to undergo DSE, Asp126 and Val155, one affected in rod formation and impaired in DSE, Val18, and two able to form rods, Lys27 and Val143, were chosen for this experiment. These residues of PapA were mutated to Tyr in the Pap operon (Lindberg et al., 1984). The results (Figures 4E and S4D) clearly show that all PapA mutants can haemagglutinate red blood cells comparably to wild-type, except Val18Tyr. Thus, whether rod formation is impaired (Asp126Tyr or Val155Tyr) or not (Lys27Tyr and Val43Tyr), all mutants are able to form pili on the bacterial surface, suggesting that impairment in rod formation is not sufficient to prevent pilus extrusion. Only Val18Tyr, which is impaired in DSE (Figure 4D), is affected in in-vivo-pilus production. It cannot be excluded that the presence of the usher mitigates any defects observed in vitro and that interface mutations may have an effect on the rate of subunit assembly in vivo.

Finally, another set of mutations was made to evaluate the biological impact of interface residues on pilus biogenesis and quaternary structure in vivo. Nine residues were selected for mutation: Thr3, Ser23, Lys50, Asn94, Lys125, Asp126, His132, Thr134, and Val155 (see Figure S4E for location and Figure S3 for interactions). Residues were mutated to Arg or Glu as indicated in Figure S4F. Among those mutants, three are at positions already investigated using AzF or Tyr: Lys50, Asp126, and Val155. All mutants were tested for pilus production in vivo by harvesting them from the bacterial surface (Hoschützky et al., 1989). Resulting pili preparations were also assessed for the structural integrity of the rod using a simple SDS-urea assay to assay the strength of the helical interaction (see Experimental Procedures). The results are presented in Figure S4F. Mutation of Thr3 to Arg did not affect pilus production on the *E. coli* cell surface, and pili were sensitive to urea treatment, indicating native-like rod formation. Interestingly, this might suggest that the staple region might not be absolutely essential in rod formation. All mutants produced PapA polymers, but the resulting filaments appear to have reduced quaternary structural stability consistent with their position in the structure. Note that the effects of mutations observed both in vivo (Figure S4F) and in vitro (Figure 4C) for the Lys50, Asp126, and Val155 mutants were the same.

### Conclusions

The structure of the PapA helical rod provides unprecedented atomic details of interactions that play essential roles in UPEC pathogenesis. It explains some of the most intriguing properties displayed by these biological fibers, i.e., their ability to uncoil and coil elastically to provide mechanical resistance to shear forces. Indeed, the rod’s quaternary structure is maintained by a network of polar interactions that would offer some resistance to forces exerted on it by the flow of urine in the urinary tract but would also progressively break as these forces increase with stronger flows. During this process, the polymer, although losing its quaternary structure, retains its integrity because of the strong, mostly hydrophobic, DSE interaction. Thus, a finely tuned interplay of interactions appears to provide the rod with mechanical properties particularly well adapted to the environment of the urinary tract. The bacterium can thus remain attached even while sustaining intense shear forces. Quaternary structure formation at the exit of the usher pore has also been hypothesized to provide the driving force for extrusion of the nascent pilus. Our data suggest that interface mutations are not sufficient to prevent pilus translocation and hence other factors might be at play. Importantly, the atomic model of the pilus rod arms us with the necessary information to answer such questions in the near future. Possible mechanisms for preventing the nascent pilus from sliding back might be afforded by the plug domain on the periplasmic side of the usher, which might act as a ratchet. Finally, this structure paves the way for the design of “coilicides” (Klinth et al., 2012), compounds and biologics that could interfere with rod formation and, thus, might greatly impair the ability of bacterial pathogens to maintain a foothold in the urinary tract.

## Experimental Procedures

### Plasmids

The construct for PapD_His_:PapA wild-type (pTRC99A) has previously been described (Verger et al., 2007). Mutants of *papA* were created to allow the incorporation of the unnatural amino acid (UAA) AzF. This was achieved by site-specifically introducing the amber codon, TAG, using the QuickChange protocol but with KOD polymerase (Merck Chemicals). *papD*_*His*_*:papA* wild-type served as the template in all mutagenesis PCR reactions. The sites chosen in PapA for AzF incorporation were Val18, Lys27, Lys50, Lys67, Thr76, Asn96, Gln106, Asp126, Val143, and Val155. A plasmid encoding the required aminoacyl tRNA synthetase/tRNA pair for AzF incorporation, pDULE2 pN3F RS (Miyake-Stoner et al., 2009), was a kind gift from Ryan Mehl (Oregon State University). The pPAP5 construct, which allows constitutive expression of the *pap* operon (pBR322), was previously described (Lindberg et al., 1984). Mutations (Val18Tyr, Lys27Tyr, Asp126Tyr, Val143Tyr, and Val155Tyr) were introduced within the *papA* gene using a QuickChange protocol (as above).

### Protein Expression and Purification

Recombinant PapD_His_:PapA wild-type was expressed in *E. coli* C600 cells (Zymo Research). Cultures were grown to OD_600_ of 0.6–0.8 in LB media before being induced using 1 mM IPTG at 20°C for 12 hr. The first step of purification involved periplasmic extraction, whereby the cells were resuspended in 20 mM Tris (pH 8.0), 150 mM NaCl, and 20% w/v sucrose and incubated for 20 min in the presence of 5 mM EDTA, 0.1 mg/ml lysozyme, 1 μg/ml DNase, and complete mini EDTA-free protease inhibitor cocktail tablets (Roche). The resulting periplasmic extract was clarified by centrifugation at 12,000 × *g* for 20 min, and the supernatant was dialysed against 20 mM Tris (pH 7.5) and 150 mM NaCl. PapD:PapA was purified using nickel affinity chromatography (HisTrap HP column, GE Healthcare) and size exclusion chromatography (Superdex 75, GE Healthcare) in 20 mM Tris (pH 7.5) and 150 mM NaCl. Proteins were concentrated using Amicon spin concentrators (3 kDa MW cutoff). All purifications were performed at 4°C.

### Generation and Purification of PapA Pilus Rods

In vitro polymerization of purified PapD:PapA complexes (wild-type and AzF-incorporated mutants) into PapA pilus rods was carried out in 400 μl reactions at 20°C for 72 hr at a concentration of 50 μM. The resulting PapA pilus rods were purified by four rounds of ultracentrifugation at 57,000 × *g*. After each cycle of ultracentrifugation, the resulting pellets were washed in 20 mM Tris (pH 7.5) and 150 mM NaCl, and final samples of purified pilus rods were resuspended in 200 μl of the same buffer. The supernatants of the first ultracentrifugation step, which contained leftover unpolymerized PapA and PapD, were retained as control samples for subsequent labeling reactions.

### Cryo-Electron Microscopy

A sample of pilus rods (5 μl) was applied to glow-discharged Quantifoil 1.2/1.3 400 mesh grids (Agar Scientific), previously covered with a 7-nm-thick layer of continuous carbon, and was incubated for 1 min. The grids were blotted and plunged into liquid ethane using a manual plunging device. Grids were stored in liquid nitrogen. The data were collected on an FEI Krios electron microscope with Titan 2.2 software, equipped with an XFEG, and operated at 300 kV. Images were collected on a Falcon II detector with a 1.1 Å pixel size and a defocus range of −1.5 to −2.7, with the beam diameter just larger than the detector (∼500 nm) using Nanoprobe mode. A total dose of 135 electrons/Å^2^ was collected with the dose equally divided among 40 frames to allow for dose fractionation.

### Cryo-EM Image Processing and Reconstruction

A total of 90 images (each 4,000 × 4,000 px) were selected that were free of drift and astigmatism and had a defocus less than 3.0 μm. The program CTFFIND3 (Mindell and Grigorieff, 2003) was used for determining the contrast transfer function (CTF), and the range used was from 1.0 to 3.0 μm. The SPIDER software package (Frank et al., 1996) was used for most subsequent steps. The CTF was corrected by multiplying each image by the theoretical CTF, both reversing the phases where necessary and improving the signal to noise ratio. The program e2helixboxer within EMAN2 (Tang et al., 2007) was used for boxing long filaments from the micrographs, and 1,277 such boxes of varying length were generated from the images. Overlapping boxes, 384 px long with a 10 px shift (∼1.5 times the axial rise of the subunit) between adjacent boxes (97% overlap), were extracted from these long filaments, yielding 56,341 segments. However, an initial map was generated using only 23,922 segments. A second, independent map was generated from the remaining 32,419 segments. The CTF determination and particle picking came from the integrated images (all 40 frames), while the segments used for the initial alignments and reconstruction came from the first 11 frames (with a dose of 37 electrons/Å^2^). The final reconstruction was generated by imposing the helical parameters found for each segment using the first 11 frames on segments containing only the first 5 frames (∼17 electrons/Å^2^) and using these for the back-projection in SPIDER. This procedure minimized both motion and radiation damage at the same time. The iterative helical real space reconstruction (IHRSR) algorithm (Egelman, 2000) was used for the helical reconstructions, starting from a solid cylinder as an initial model. The amplitudes of the final volume were corrected for the CTF by dividing by the sum of the squared CTFs, since each image had been multiplied by the CTF twice: once by the microscope and once computationally when phases were corrected.

### Model Building and Refinement

Model building began with a docked model of the PapA subunit-subunit (PDB: 2UY6). Models were docked using Chimera’s “dock into density” tool. The strand insertion (not present in the crystal structure) was modeled by taking residues 10–19 from the crystal structure and inserting it in the adjacent subunit. However, this left nine N-terminal residues and three residues connecting the inserted strand with the adjacent subunit unmodelled. To rebuild these residues, we used an enumerative rebuilding strategy in Rosetta. This approach iteratively samples short, three-residue segments of backbone. By only considering three-residue segments, we may completely explore the space of backbone conformations given each three-amino-acid segment. As our model is grown, up to 50 solutions are stored. Following each rebuilding step, models are refined with a low-resolution force field (Song et al., 2013). The density data are used to filter solutions inconsistent with density data (by throwing out solutions with density agreement significantly worse than the best seen over the same stretch of backbone). Additional filters ensure that models stored after each iteration are sufficiently different from one another. Sampling these terminal conformations revealed good convergence of the top-scoring models.

Following rebuilding of these segments, all-atom coordinates and B-factor refinement of the symmetric full-length model against the experimental density data was carried out in Rosetta, using a previously described protocol (DiMaio et al., 2015). A total of 600 models were sampled. Following refinement, the lowest-energy 20 models were selected and compared to an independent reconstruction. The 10 models with best agreement to this reconstruction show tight convergence, with an average Cα RMSD to the best model of just 0.93 Å and an average all-atom RMSD of 1.79 Å, with most of the deviation in solvent-exposed loops.

The agreement of the model to an independent reconstruction (hereafter, map2) showed some potential overfitting between model and map, as the Fourier shell correlation (FSC) curve between the model and map used for fitting appeared worse than that used for evaluation. However, when the refinement was repeated with the maps reversed, we see the opposite trend: the agreement to the independent map (now map1) was virtually identical to that of the map used for fitting. This suggests that the quality of map2 is lower than that of map1. Finally, the model refined against map1 (the higher-quality map of the two) was then compared against the full reconstruction, yielding the FSC curve shown in Figure S1D. This shows an FSC = 0.25 at a resolution of 3.8 Å, which is in agreement with the observed features present in the density maps, including individual strand separation and visible density for bulky side chains.

### AzF Incorporation into PapA

Mutant plasmids of PapA, for the incorporation of AzF, were co-transformed into *E. coli* C600 cells along with pDULE2 pN3F RS. Protein expression with site-specific AzF incorporation was achieved by growing cultures in Luria-Bertani (LB) media in the presence of 1 mM AzF (SynChem). All other aspects of protein expression and purification were the same as for PapD_His_:PapA wild-type.

### Labeling of PapA Pilus Rods with Alexa 647

Purified samples of PapA pilus rods (wild-type and AzF-incorporated mutants) were labeled with click-it Alexa Fluor 647 DIBO alkyne (Life Technologies) to assess the accessibility of each chosen site of AzF incorporation in the quaternary structure of the PapA pilus rod (Chin et al., 2002, Miyake-Stoner et al., 2009). Samples of purified pilus rods were incubated with an estimated 10-fold molar excess of click-it Alexa Fluor 647 DIBO alkyne in 100 μl reactions for 1 hr at 20°C. Control reactions, whereby unpolymerized PapA and AzF-incorporated PapA mutants (supernatants after first ultracentrifugation step) were labeled, were performed in parallel in 40 μl reactions under identical conditions. Protein concentrations were 5–20 μM for all labeling reactions. Excess unreacted Alexa Fluor 647 was removed by two further rounds of ultracentrifugation and by dialysis for the purified pilus rods and unpolymerized control samples, respectively. Reactions were subsequently analyzed by SDS-PAGE, using 4%–12% NuPAGE gradient gels with MES buffer (Life Technologies). Samples prior to Alexa Fluor 647 labeling but identical in every other respect (including concentration) were analyzed on separate gels and stained with Sypro Ruby protein gel stain (Life Technologies). Gels were imaged using a FLA-3000 fluorescent image analyzer (Fujifilm) using a 633 nm (for Alexa fluor 647) and 473 nm (for Sypro Ruby) scanning wavelength, and fluorescent band intensities were quantified using the ImageQuantTL software (GE Healthcare). Identical loading controls were included on all gels to allow normalization of the fluorescent intensities from different gels. In addition, the intensity of the PapD band was used as an internal control to normalize the intensity of PapA between pre- and post-Alexa-labeled gels.

To assess pilus rod formation, the Sypro Ruby signals were normalized to the wild-type signal (100%) and plotted. To assess labeling efficiency, the resulting ratios of Alexa fluor 647 to Sypro Ruby signals were compared between purified pilus rod samples and the equivalent non-piliated controls and served as an indication of the surface accessibility of the fluorescent dye in the context of the quaternary structure of pilus rods. If PapA in pilus rods labeled to the same extent as in the unpolymerized control form, the final ratio would be 1.0.

### Assessment of DSE Reactions in PapD:PapA Mutants

Samples of purified PapD_His_:PapA wild-type, Val18AzF, Asn96AzF, Asp126AzF, and Val155 AzF were left to polymerize at 20°C for 16 hr at a concentration of 38 μM. These samples (100 μl) were loaded consecutively onto a Superdex 200 10/300 increase column (GE Healthcare) to assess whether PapA has undergone DSE. To unambiguously identify the species responsible for each peak, a sample of PapD:PapA Val18AzF was run on a SEC-MALS instrument (Wyeth) also using a Superdex 200 10/300 increase column (GE Healthcare) and the molecular weight of each peak was derived using the manufacturer’s software.

### Negative Stain Electron Microscopy

Samples of ultracentrifuge-purified PapA pilus rods (wild-type and AzF-incorporated mutants; 5 μl) were applied to carbon-coated and glow-discharged copper grids (Agar Scientific) and incubated for 1 min. After incubation, the samples were blotted, washed with two drops of water, blotted once more, and stained with 2% w/v uranyl acetate. Image frames were acquired with a Gatan CCD camera (2,000 × 2,000 px) on a Tecnai T12 electron microscope (FEI) operated at 120 kV.

### Haemagglutination of P Pili-Expressing Cells

Plasmids harboring either wild-type or mutant versions of PapA in the Pap operon (pPAP5; see above) were transformed into HB101 *E. coli* cells (Promega) and cultured on tryptic soy agar (Sigma-Aldrich) plates. After growth at 37°C, bacterial cells were harvested from plates using PBS ([pH 7.4]; Sigma-Aldrich), and the cell density was normalized by measuring the optical density (OD) at 600 nm. A 2-fold serial dilution of bacterial cells was established in a 96-well, V-bottomed plate (Thermo Scientific), and bacteria were incubated with 10% rabbit red blood cells (Stratech; previously washed in PBS) for 1 hr at 4°C. Untransformed HB101 (HB101 alone) and PBS served as negative controls for this experiment, while pPAP5 wild-type served as the positive control.

### Purification of In Vivo-Produced Pili

For pili production, C600 *E. coli* cells were co-transformed with a plasmid harboring the Pap operon but lacking the gene for PapA (pFJ3) (Jacob-Dubuisson et al., 1993) and with a second plasmid harboring PapA wild-type or mutants (pTRC99A). Mutations were introduced into pTRC99A using a QuickChange protocol. Pili production was induced by plating cells on tryptic soy agar plates supplemented with IPTG and cells were harvested after growth at 37°C. Pili were detached from cells by heat treatment at 65°C for 1 hr (Hoschützky et al., 1989) and separated from *E. coli* cells by pelleting the depiliated bacteria by centrifugation.

### Urea-Induced Uncoiling of In Vivo-Produced Pili

Wild-type pilus helical rods are resistant to denaturation by SDS and heating but can be depolymerized in the presence of high concentrations of other denaturants such as urea (Hoschützky et al., 1989, Karch et al., 1985). To test the effect of mutations on the strength of helical interactions, aliquots of pilus preparations (described above) were mixed in an SDS sample buffer to a final concentration of 2% (w/v) SDS, 40 mM Tris (pH 6.8), and 1 mM beta-mercaptoethanol with or without 4.5 M urea and incubated at 95°C for 15 min. These samples were then allowed to cool to room temperature and glycerol was added to a final concentration of 15%. The samples were analyzed by SDS-PAGE and PapA bands were detected by Coomassie staining. The intensity of PapA monomer bands in lanes of mutants treated with and without urea was assessed to determine the effect on helical stability.

## Author Contributions

M.K.H. designed, performed, and analyzed all biochemical experiments, made mutations in pPAP5, performed haemagglutination assays with help from A.R., made figures and wrote the paper. A.R., C.R., and M.K.H. collected NS-EM pictures. A.R. and M.U. helped set up the cryo-EM. B.F. and F.D. built the model. K.D. and S.J.H. made mutants described in Figure S4F and tested their ability to elaborate pili and form rods. E.H.E. solved the structure, made figures, and wrote the paper. G.W. supervised the work, made figures, and wrote the paper.

## Accession Numbers

The accession number for the EM map reported in this paper is EMDB: EMD-3222. The accession number for the model coordinates reported in this paper is PDB: 5FLU.

## Supplemental Information

Document S1. Article plus Supplemental Information
